# Design and synthesis of the superionic conductor Na_10_SnP_2_S_12_

**DOI:** 10.1038/ncomms11009

**Published:** 2016-03-17

**Authors:** William D. Richards, Tomoyuki Tsujimura, Lincoln J. Miara, Yan Wang, Jae Chul Kim, Shyue Ping Ong, Ichiro Uechi, Naoki Suzuki, Gerbrand Ceder

**Affiliations:** 1 Department of Materials Science and Engineering, Massachusetts Institute of Technology, Cambridge, Massachusetts 02139, USA; 2 Samsung R&D Institute Japan, Minoh Semba Center Building 13F, Semba Nishi 2-1-11, Minoh, Osaka 562-0036, Japan; 3 Samsung Advanced Institute of Technology—USA, 255 Main Street, Suite 702, Cambridge, Massachusetts 02142, USA; 4 Materials Sciences Division, Lawrence Berkeley National Laboratory, Berkeley, California 94720, USA; 5 Department of NanoEngineering, University of California San Diego, La Jolla, California 92093-0448, USA; 6 Department of Materials Science and Engineering, University of California Berkeley, Berkeley, California 94720, USA

## Abstract

Sodium-ion batteries are emerging as candidates for large-scale energy storage due to their low cost and the wide variety of cathode materials available. As battery size and adoption in critical applications increases, safety concerns are resurfacing due to the inherent flammability of organic electrolytes currently in use in both lithium and sodium battery chemistries. Development of solid-state batteries with ionic electrolytes eliminates this concern, while also allowing novel device architectures and potentially improving cycle life. Here we report the computation-assisted discovery and synthesis of a high-performance solid-state electrolyte material: Na_10_SnP_2_S_12_, with room temperature ionic conductivity of 0.4 mS cm^−1^ rivalling the conductivity of the best sodium sulfide solid electrolytes to date. We also computationally investigate the variants of this compound where tin is substituted by germanium or silicon and find that the latter may achieve even higher conductivity.

## Abstract (web summary)

Solid electrolytes are an attractive alternative to the flammable organic solvents typically used in intercalation batteries. Here, the authors report the computation-assisted discovery and synthesis of Na_10_SnP_2_S_12_, a sodium electrolyte with room temperature conductivity of 0.4 mS cm^−1^.

## Graphical abstract

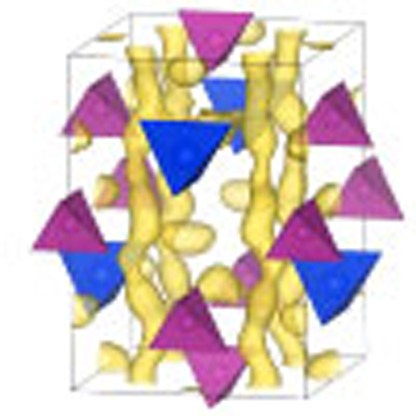

The energy density and cycle life of intercalation batteries has made them the dominant technology in all manner of applications, from consumer electronics to electric vehicles and high-performance batteries in commercial aircraft. A potential key application of sodium (Na)-ion battery technology is grid-scale energy storage due to its lower cost relative to lithium (Li)-ion. In addition to sodium being considerably more abundant than Li, Na-ion batteries have the advantage of a broader range of available cathode materials since many layered Li-transition metal oxides show improved performance in their sodium versions^1^,^2^,^3^. In addition, many high capacity Na-cathodes do not contain cobalt, an expensive and scarce component of many commercial Li-ion cathodes. The choice of electrolyte for Na-ion systems is not as well established as for Li-ion, but the vast majority of electrolytes in development are based on organic solvents^4^. These suffer from the same flammability concerns as their counterparts in lithium batteries, and are exacerbated by the presence of a much more reactive metal. In addition, heat dissipation properties of the battery are worsened by the size of installations required for grid storage, and thermal runaway is therefore an even greater concern than at smaller scales. The creation of low-temperature sodium-solid electrolytes would go a long way towards the development of safe solid-state Na-ion batteries, free of flammable solvents.

Despite much work in the area, no anode materials for sodium ion systems have been found that can match the conductivity, energy density and price of graphite anodes in Li-ion batteries. Though hard carbon anodes have been shown to reversibly intercalate sodium^5^, capacity is very low compared with that allowed by intercalation to LiC_6_ (ref. 6). Solid electrolytes may also suppress dendrite formation, enabling the use of sodium metal anodes and improving battery capacity considerably.

Na-solid electrolytes have been commercialized in high-temperature batteries such as β-alumina for sodium–sulfur (NAS) batteries^7^, yet few materials with high conductivities at low temperature have been reported. Conductivities over 1 mS cm^−1^ have been shown in NASICON-type oxide crystals^8^,^9^, but processing of these materials at high temperatures (typically >1,000 °C (refs 7, 10)) is required to reduce grain boundary resistance, which is incompatible with typical cathode materials and complicates battery fabrication. In both sodium and lithium systems, thiophosphate materials are promising candidates as solid electrolytes as they are soft and can be incorporated into batteries by cold pressing without requiring high-temperature sintering. In 2011, Kamaya *et al*. reported the synthesis of Li_10_GeP_2_S_12_ (LGPS), a tetragonal structure within the Li_3+*x*_Ge_*x*_P_1−*x*_S_4_ thiophosphate system that achieves 12 mS cm^−1^ conductivity at room temperature^11^, and in 2014 Seino *et al*. reported a conductivity of 27 mS cm^−1^ with a glass-ceramic electrolyte composed of Li_7_P_3_S_11_ crystals precipitated from a glass^12^. There has been relatively less experimental work done on sodium systems, though recently the cubic phase of Na_3_PS_4_ has been reported to have conductivity as high as 0.46 mS cm^−1^, and has been used in an all-solid-state battery^13^,^14^. Similar to lithium materials, Si doping has recently been used to increase defect concentrations in Na_3_PS_4_, resulting in a conductivity of 0.7 mS cm^−1^ (ref. 15). For construction of solid-state cells, low-strain electrodes^16^,^17^ are also important to minimize delamination of the electrolyte, especially when using harder electrolyte materials such as oxides.

*Ab initio* calculation of material properties can rapidly speed up the search for new solid electrolyte materials, allowing prediction of as-yet-undiscovered materials through calculations of phase diagrams^18^ and evaluation of the diffusivity of carefully controlled structures, which normally can only be achieved experimentally after a long process of synthesis optimization. Even when new hypothetical materials show similarity to existing materials, chemical intuition alone cannot reliably determine which chemical modifications will result in experimentally realizable structures, for example, the high conductivity lithium garnets^19^ have no direct sodium analogues. *Ab initio* calculations have shown remarkable accuracy in predicting the properties of Li-solid-state conductors^11^,^20^,^21^,^22^,^23^,^24^. Here we present computational predictions of three Na-ion conductors crystallizing in the high-conductivity tetragonal structure and with conductivities equalling and exceeding the current best performing materials. We also show experimental results confirming our prediction of the Na_10_SnP_2_S_12_ material, with experimentally measured conductivity and activation energy in excellent agreement with the *ab initio* result. In addition to rigorously probing the energetics and hence feasibility of synthesis of these materials, computational techniques are able to give an indication of their performance. They are thus able to focus experimental efforts on systems with a high probability of success. These results highlight the predictive nature of first principles calculations—in addition to explaining difficult to observe phenomena, they can be used to discover compounds with extraordinary physical properties.

## Results

### General considerations

Using first principles computation, we evaluate three key properties of the tetragonal phases of Na_10_MP_2_S_12_ (M=Si, Ge, Sn) to determine their suitability as a solid-state electrolyte materials: (1) we determine the Na^+^ conductivity and its activation energy from *ab initio* molecular dynamics (AIMD) simulations, (2) using high-throughput computations and structure prediction methods, we comprehensively calculate the ground-state phase diagram of each system to gauge the stability and synthesizability of each compound and (3) we extract the electrochemical anodic and cathodic stability limits from the grand canonical equilibrium at various potentials similar to the approach described in an earlier work^25^. Based on this data, we then proceeded to synthesize and test Na_10_SnP_2_S_12_.

### Ground-state energy calculations

**Figure 1:**
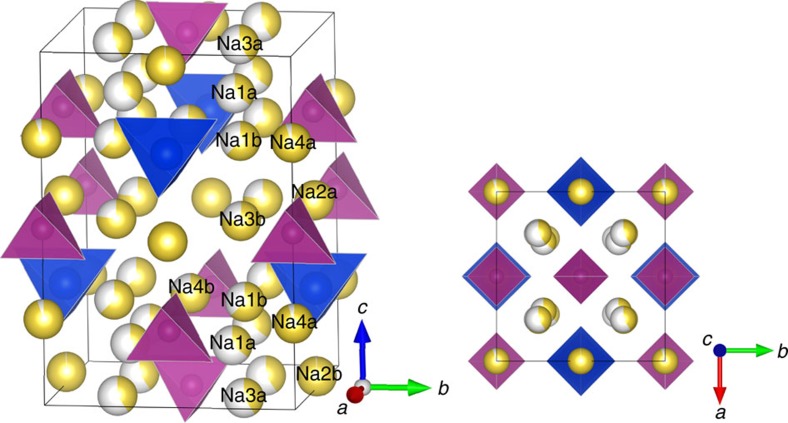
Structure of Na_10_SnP_2_S_12_ from DFT calculations. Sodium occupancies are calculated from 600 K AIMD simulation (see Methods). All ground-state NMPS structures share this M/P ordering, which reduces the symmetry from the *P*4_2_/*nmc* space group to 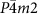, separating each Na-site into two symmetrically distinct but similar sites marked as *a* and *b*. PS_4_ tetrahedra are marked in purple, SnS_4_ tetrahedra in blue and Na-sites in yellow. The ground-state Na-ordering is shown in Supplementary Fig. 2.

Since there is typically considerable cation site disorder in these conductors, we used an electrostatic energy criterion to pre-sceen Na/Vacancy orderings on the experimentally reported structure of LGPS^26^. For each of the three symmetrically distinct M/P orderings and for full and half Na4 site occupancy, we relaxed the structures of the lowest electrostatic energy arrangements using density functional theory (DFT), taking the lowest energy of these as the 0 K enthalpy and structure. The structure of Na_10_MP_2_S_12_ (NMPS) can be described as consisting of three symmetrically distinct chains of cations oriented parallel to the *c*-axis (Fig. 1). At unit cell coordinates *x*=0.25, *y*=0.25, tetrahedral Na sites (Na1, Na3) form a chain of partially occupied edge-sharing sites. At *x*=0, *y*=0.5 there is an edge-sharing chain of alternating Na_oct_ and (M/P)_tet_ sites. At *x*=0, *y*=0, a similar chain but with a vacancy instead of M cation and more distorted Na_oct_ site is present (with repeat unit Na_oct_-P_tet_-Na_oct_-Vac_tet_). The *ab initio* MD results will demonstrate that the (Na1, Na3) chains carry most of the Na conductivity with occasional crossover through the Na sites in the chain at *x*=0, *y*=0. The ground state M/P ordering, which is found to be shared among all studied chemistries, is shown in Fig. 1 and the ground-state Na-ion arrangement (*C*222 space group) in Supplementary Fig. 2.

### *Ab initio* molecular dynamics

**Figure 2:**
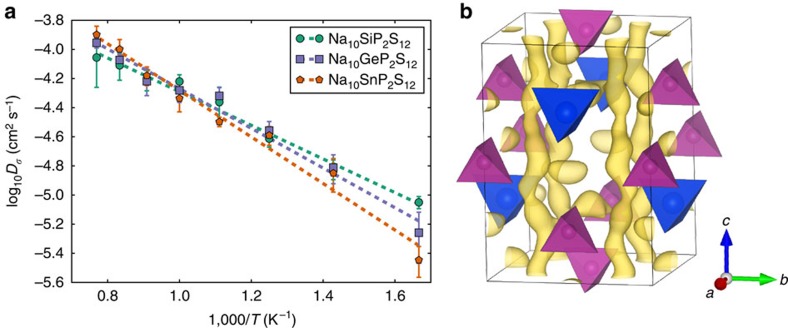
DFT computed diffusivity of Na_10_SnP_2_S_12_. (**a**) Na-diffusivity in Na_10_SiP_2_S_12_, Na_10_GeP_2_S_12_ and Na_10_SnP_2_S_12_ from AIMD simulation. Dashed lines are Arrhenius fits to the data, and error bars are standard error of the mean. (**b**) Na-ion probability density isosurface (yellow) of Na_10_SnP_2_S_12_ from 600 K AIMD simulation. SnS_4_ tetrahedra are marked in blue, PS_4_ tetrahedra in purple.

The Na-ionic conductivity (*σ*), and activation energy (*E*_a_) were determined from AIMD simulations between 600 and 1,300 K and extrapolated to room temperature. Ionic conductivity is calculated from AIMD through the intermediate calculation of *D*_*σ*_, which has the units of a diffusivity but takes into account correlations between Na-ions (see Methods). The results are shown in Fig. 2a, and compared with similar Li compounds in Table 1. The self diffusivity (*D*_self_) of the Na-ions was also calculated for comparison, with results included in Supplementary Table 1 and Supplementary Fig. 1. For both the Li and Na materials, activation energy slightly increases as M changes from Si→Ge→Sn. Somewhat surprisingly, given the size difference between Na and Li ions, Na and Li materials have similar activation energies, resulting in high room temperature conductivities particularly for the Ge and Si materials, which are predicted to have room temperature conductivities comparable to those of organic electrolytes^4^. Our result for Na_10_GeP_2_S_12_ is similar to the result of ref. 27. These conductivities are more impressive, given that they are entirely due to Na^+^ motion, and so the transference number is equal to 1. The degree of cooperativity of ionic motion is described by the Haven ratio *H*_r_ (ref. 28), which we calculate from the ratio of *D*_self_ to *D*_*σ*_. This value is calculated to be ∼0.56 in all of our simulations, which is slightly smaller than that observed experimentally for the lithium versions of these materials^22^,^23^, indicating a larger degree of cooperative motion.

### Phase diagrams and stability limits

**Figure 3:**
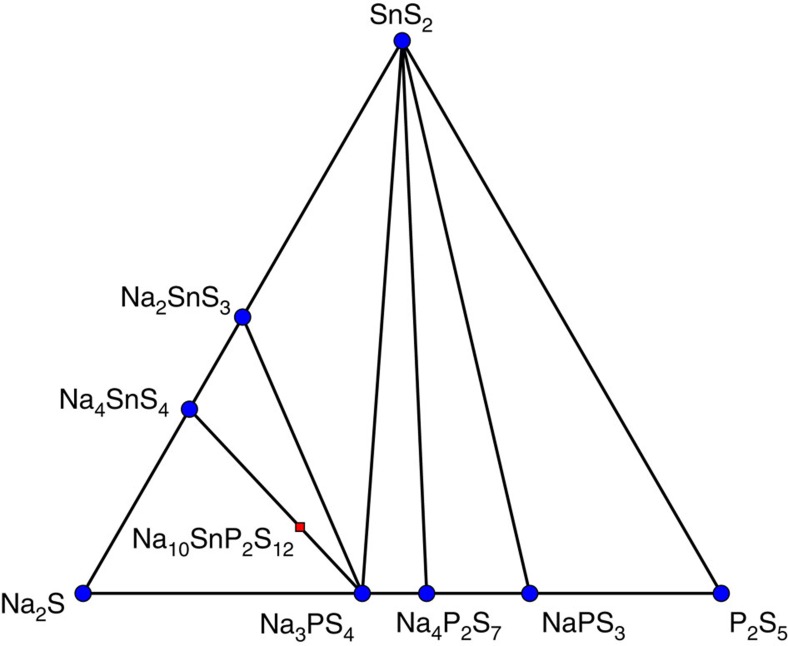
Na-Sn-P-S phase diagram. Pseudo-ternary 0 K Na-Sn-P-S phase diagram constructed from DFT energy calculations, with location of Na_10_SnP_2_S_12_. Stable phases marked with blue dot.

To determine the feasibility of synthesizing these high conductivity tetragonal phases of Na_10_MP_2_S_12_ (M=Si, Ge, Sn), we used DFT to evaluate the energies of materials and generate their respective quaternary phase diagrams. To obtain appropriate competing phases in the quaternary phase diagrams, we calculated the energy of a very large number of compounds in their relevant chemical spaces, including all known materials present in the Inorganic Crystal Structure Database (ICSD)^29^ containing some or all of the four elements, all relevant materials derived from substituting sodium for lithium in all ICSD materials and the Li_*x*_P_*y*_S_*z*_ structures compiled by Lepley *et al*.^30^. To further improve the coverage of these chemical spaces, we also applied the data-mined substitution methodology of Hautier *et al*.^18^ to predict possible structures from a broader range of chemistries in the ICSD. The 0 K phase diagram for Na-Sn-P-S is shown in Fig. 3. Na-Ge-P-S and Na-Si-P-S phase diagrams are available in Supplementary Fig. 3.

No quaternary ground states are found in any of the three systems. Decomposition energy (*E*_decomp_) to the equilibrium ground-state structures is calculated using the convex hull method implemented in pymatgen^31^ and is shown in Table 2, and compared with their lithium counterparts. For example, the stability of the Na_10_SnP_2_S_12_ phase is given by the calculated enthalpy of the decomposition reaction Na_10_SnP_2_S_12_→2 Na_3_PS_4_+Na_4_SnS_4_. Even though all the considered electrolyte structures show a small driving force at 0 K to decompose to (Li/Na)_4_MS_4_ (M=Si, Ge, Sn) and (Li/Na)_3_PS_4_, this is similar to the Li-analogues that have similar decomposition energies, and have all been synthesized^11^,^21^,^23^,^24^. We expect high configurational entropy on the cation sites to result in their stabilization at moderate temperatures. An approximation of this entropy, neglecting the ion–ion interactions, can be obtained using the formula *S*=−*k*_B_∑_*i*_*p*_*i*_ ln *p*_*i*_, where *k*_B_ is the Boltzmann constant, *p*_*i*_ is the probability of each state (occupied or unoccupied) and the sum is over all states for each site. Using a value of 50% occupancy of the Na-atoms in the edge-sharing *c*-axis chains and 50% M/P occupancy (28 sites with 50% occupancy per 50 atom unit cell) yields a value of 0.0334, meV K^−1^ per atom, which at 300 K already would stabilize the Sn and Ge compositions. This is an upper bound on the configurational entropy but vibrational entropy, particularly the soft phonon modes of the diffusing ions, is also expected to contribute to the structure's stabilization. Table 2 also shows the calculated anodic and cathodic stability limits evaluated from the chemical potentials of Na at which the compound decomposes, following the methods of ref. 25. Since these materials by our calculations are metastable at 0 K, we instead consider the potentials at which the ground-state materials equilibrium becomes unstable, for example, for Na_10_SnP_2_S_12_, when either Na_4_SnS_4_ or Na_3_PS_4_ becomes unstable.

When the chemical potential (voltage) of the alkali is below (above) the stable region (as can be experienced at the cathode interface during charging), the ion and its associated electron is pulled from the electrolyte, which decomposes into a mixture of sulfides and elemental sulfur (for example, Na_4_SnS_4_ decomposes to S and Na_2_SnS_3_ above 1.82 V versus Na metal, and S and SnS_2_ above 2 V). In contact with the anode (cathodic limit), the Li/Na metal may reduce the metal or phosphorus in the electrolyte, potentially leading to electron conductivity through the electrolyte if this reaction continues without passivation. The cathodic limit for Na and Li compounds is set by the partial reduction of phosphorus to form Na_2_PS_3_, and the calculated cathodic stability is thus unaffected by the choice of metal (M) cation. The potentials at which the metal cation is fully reduced by the alkali are also listed in Table 2, and indicate the potential at which the decomposition reaction is no longer passivating. The shift in the stability window between the Na and Li materials is due to the differing reduction potentials of the alkali metal. Previous DFT studies have shown that this reduction reaction can be passivated in some systems by the formation of a thin layer of Li_2_S (ref. 30), though in practice insulating barrier coatings are typically employed at the anode/cathode interfaces^11^,^32^,^33^. The anodic voltage stability limit is set primarily by the reaction energy of the alkali metal with elemental sulfur, though in compounds with highly negative enthalpies of mixing from the binary sulfides the stability range is extended slightly. This effect is small in the considered electrolyte materials, with the anodic stability only changing on the order of 0.1 V between materials with different (M) cations.

### Synthesis and experimental verification

**Figure 4:**
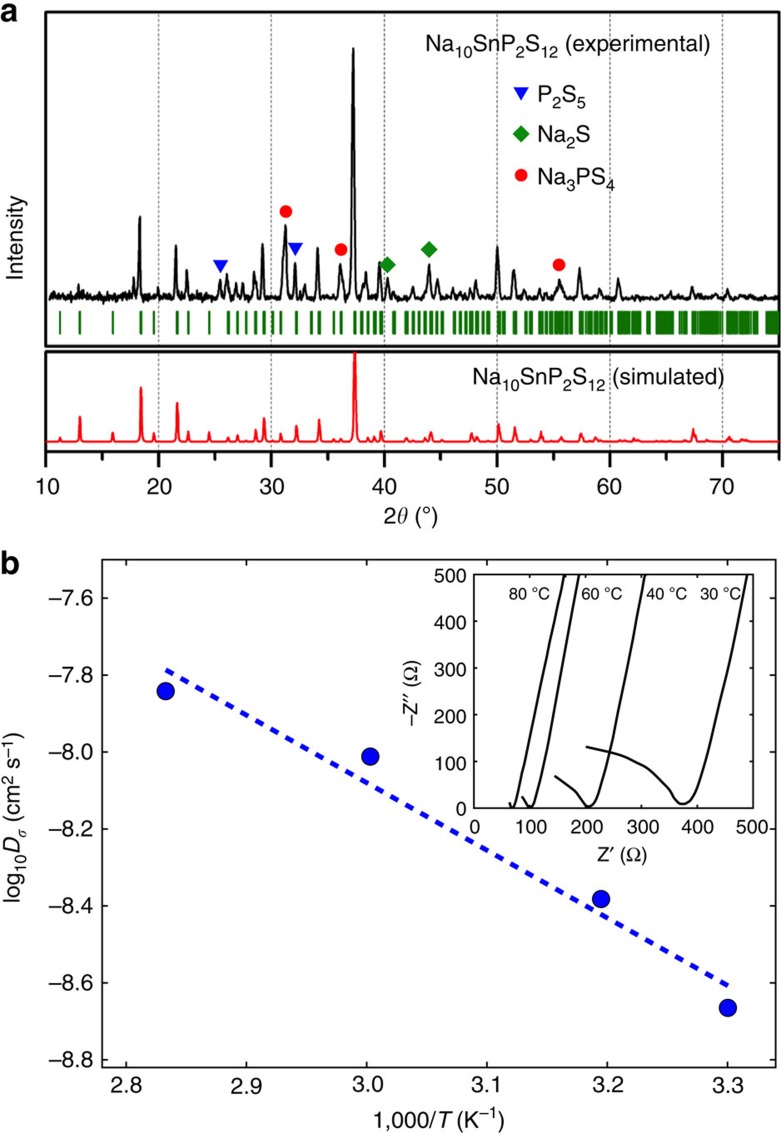
Experimental crystal structure and diffusivity of Na_10_SnP_2_S_12_. (**a**) Experimental and simulated XRD patterns of Na_10_SnP_2_S_12_, showing small amounts of recrystallized P_2_S_5_, Na_3_PS_4_ and Na_2_S. (**b**) Diffusivity calculated from experimentally measured ionic conductivity versus temperature. Dashed line is an Arrhenius fit to the data. (inset) Electrochemical impedance spectroscopy measurements.

In validation of our computational predictions, we report successful synthesis of Na_10_SnP_2_S_12_, which was chosen due to its low materials cost and *E*_decomp_ of 7.1 meV per atom, which is lower than comparable materials that have been synthesized. Na_10_SnP_2_S_12_ was prepared from the binary sulfide phases (see Methods), under a range of cooling rates. The lattice volume and conductivity of the synthesized phase increase as the cooling rate is lowered (Supplementary Fig. 4), with the highest conductivity achieved by cooling from 700 °C over 99 h. To compare the experimental XRD pattern with that predicted from DFT calculation, we used the Na and Sn/P site disordered structure with positions and fractional occupancies of each site generated from *k*-means clustering of Na-position data from the 600 K AIMD simulation as a starting point for powder XRD simulation of the structure. Comparison of the simulated and experimental XRD patterns is shown in Fig. 4a. The obtained material is predominantly the expected tetragonal Na_10_SnP_2_S_12_, with small amounts of P_2_S_5_, Na_3_PS_4_, primarily in the tetragonal α-phase as indicated by the peak splitting at 31 and 36 degrees^13^, and Na_2_S, which formed during the slow cooling. At faster cooling rates, these impurity phases do not form but the resulting material has lower conductivity due to the lower lattice volume. The change in the lattice volume and conductivity is likely a result of the structure in the slow cooled sample having a higher ratio of Sn to P, since the observed impurities contain no Sn. Similar dependency of conductivity and lattice volume on this ratio are seen in the lithium systems^34^. The low conductivity of the impurity phases the slow-cooled sample are expected to reduce the measured conductivity by reduction in the effective cross-sectional area. The strong relation between lattice volume and conductivity also support the conductivity measured in the slow-cooled sample being that of Na_10_SnP_2_S_12_.

The intensities of the 011 and (110 and 002) reflections, producing XRD peaks at 12 and 16 degrees, vary as a function with cooling rate, but are not strongly correlated with conductivity. Supplementary Fig. 5 shows the XRD spectrum of a quenched sample in which these low-angle peaks are more clearly visible. The variation in these peak intensities may be caused either by slight disorder between the **P**_tet_, **M**_tet_ and **Vac**_tet_ sites, or by changes in average size of the (Sn/P)S_4_ tetrahedra from slight compositional variation.

Considering that AIMD simulations were performed at elevated temperatures and extrapolated to experimental conditions, the conductivity predicted from these simulations is in remarkable agreement to our experimental electrochemical impedance spectroscopy results (Fig. 4b). We predicted a room temperature conductivity of 0.94 mS cm^−1^ with activation energy of 317 meV, while experimentally Na_10_SnP_2_S_12_ shows a conductivity of 0.4 mS cm^−1^ with an activation energy of 356 meV.

## Discussion

Na_10_SnP_2_S_12_ is a remarkably good ionic conductor; its room temperature conductivity of 0.4 mS cm^−1^ is comparable to the best performing sulfide electrolyte to date—cubic Na_3_PS_4_, which achieves conductivities of between 0.2 and 0.7 mS cm^−1^ depending on doping and processing conditions^13^,^14^,^15^. These thiophosphate electrolytes benefit from improved processability relative to the oxide β-alumina and NASICON-based compounds, which can have higher conductivities but require high-temperature sintering, making them difficult to incorporate into room temperature batteries. To evaluate the potential for even better conductors in this family of compounds, we investigate in more detail the conductivity mechanism in these compounds and the effect of the main group metal (Si, Ge, Sn) on it.

From our DFT calculations, we see that the activation energy for Na diffusion in the NMPS materials shown in Fig. 2a increases as the ionic radius of the (M)etal in the compound increases, with *E*_a_^Si^<*E*_a_^Ge^<*E*_a_^Sn^. This trend is also seen in activation energies for the Li conductors, both in experimental and DFT studies (Table 1). This is surprising since often the activation energy barrier between adjacent sites in a structure decreases as the size of the anion framework increases. In the NMPS conductors, however, the lattice parameter differences are small (<1%, Supplementary Table 2), and the activation energy actually increases as the cell volume increases. The valence of the other cations near the transition state has been pointed to as an important factor as it can increase the activation energy by strong repulsion of the alkali in the activated state^35^,^36^, but this is unlikely to play a role here as Si, Ge and Sn all have valence 4+. Hence, because of their similar volume and cation valence, these three compounds form a good data set to evaluate potentially more subtle chemical influences on the conductivity. To understand the somewhat counterintuitive result, we examine the diffusion paths and site occupancies in each compound as a measure of the free-energy landscape of the structures.

From the AIMD Na-ion trajectories, we calculate the Na-ion probability density, defined as the time-averaged Na-ion occupancy, allowing visualization of the Na-ion diffusion mechanism. The probability density from AIMD simulation of Na_10_SnP_2_S_12_ at 600 K in Fig. 2b is representative of all of our AIMD simulations, and shows that the majority of the Na diffusion occurs within the *c*-axis chain of partially occupied Na sites at *x*=0.25 and *y*=0.25, with some crossover between these channels. These results are in good qualitative agreement with the highly anisotropic Li sites seen in previous spectroscopic studies on LGPS^11^,^26^.

**Figure 5:**
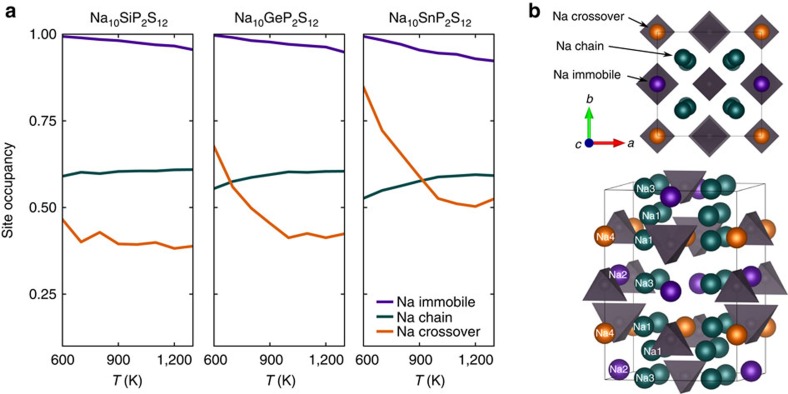
Na-site occupancy analysis of NMPS structures. (**a**) Occupancy of Na sites in Na_10_SiP_2_S_12_, Na_10_GeP_2_S_12_ and Na_10_SnP_2_S_12_ from AIMD simulation between 600 and 1,300 K, after imposing *P*4_2_/*nmc* spacegroup operations. The site occupancies in the Na-chain (Na1 and Na3) have been combined for clarity. (**b**) Illustration of the Na-chain, Na-crossover and Na-immobile sites. SnS_4_ and PS_4_ tetrahedra (grey), all spheres are Na-sites.

The Na-site occupancies of the three materials as a function of simulation temperature are shown in Fig. 5. *P*4_2_/*nmc* spacegroup operations are applied to the Na-positions before analysis to undo the splitting of Na sites caused by the M/P ordering and shown in Fig. 1. Trends in occupancy are similar for Na-sites that are part of the same *c*-axis cation chain, again confirming a flat energy landscape and high mobility along it. These Na-ion diffusion pathways are connected to each other through the Na4 (Na-crossover) sites, which are part of the Na_oct_-P_tet_-Na_oct_-Vac_tet_ chain along the *c*-axis at *x*=0, *y*=0. The Na-sites in the fully occupied Na_oct_-P_tet_-Na_oct_-M_tet_ *c*-axis chain at *x*=0, *y*=0.5 have low energy and high occupancy, and are labelled as Na-immobile sites in Fig. 5 as they are not expected to contribute strongly to diffusion at low temperatures.

At high temperatures, the occupancies of each Na-site are almost identical across the three chemistries, indicating that they are dominated by entropic effects and not by the specific enthalpic differences between the compounds. At low temperatures, relative occupancies are more dependent on differences in site enthalpy. Considering first the Sn material, the occupancy of the Na-crossover sites dramatically increases as temperature is reduced, indicating that the enthalpy of the Na-crossover sites is significantly lower than the Na-chain sites. In contrast to Na_10_SnP_2_S_12_, occupancy of the Na-crossover sites in the Si material is relatively unaffected by temperature, indicating minimal site enthalpy difference between the Na-chain and Na-crossover sites. The behaviour of occupancies in Na_10_GeP_2_S_12_ is between these two extrema.

The diffusivity of Na-ions is determined primarily by the smoothness of their free-energy landscape. In materials where atoms can be trapped in very low-energy minima, activation energy for moving between these sites is increased, and thus diffusivity is reduced. The trends in Na-crossover site energy correlate well with the activation energies observed in simulation and explain why Na_10_SiP_2_S_12_ has the highest predicted conductivity. At low *T*, the energy of the Na in the chain and crossover sites are almost equal, allowing Na to migrate in three dimensions with a very low barrier.

The good correspondence between the simulated and experimental results highlight the value of DFT as a predictive tool for the identification of new electrolyte materials. In this work, we focused synthesis efforts on the Sn material due to its affordability relative to the Ge version as well as its low *E*_decomp_ of 7.1 meV per atom, which is lower than comparable materials that have been synthesized. In LGPS and related lithium electrolytes, contact with the highly reducing lithium metal or graphite anode can cause electrolyte decomposition by reduction of the transition metal. For these sodium electrolytes this may be less of a concern due to the lower reduction potential of sodium. These newly predicted materials may also prove to be more stable in battery applications than cubic Na_3_PS_4_ material, since decomposition of Na_10_MP_2_S_12_ requires diffusion of high-valent cations to form Na_3_PS_4_ and Na_4_MS_4_, in contrast to cubic Na_3_PS_4_, which can convert to a low conductivity tetragonal phase^37^ at the same composition. The conductivity of the new Na_10_SnP_2_S_12_ electrolyte rivals that of the best known sulfide sodium-conductors, and the predicted Ge and Si materials, if confirmed, have the potential to surpass the conductivities of all known Na-electrolytes in a system much more compatible with all solid-state battery fabrication than NASICON-based and other oxide electrolytes.

In this work, we used first principles calculation to predict the existence of several new high-performance sodium electrolyte materials, with excellent agreement to subsequent experimental results. This marks the first use of computational prediction to design novel sodium electrolytes. The resulting Na_10_SnP_2_S_12_ electrolyte, with a conductivity of 0.4 mS cm^−1^ at room temperature and activation energy of 0.35 eV, rivals the best known sulfide sodium-electrolytes and our predicted materials have the potential to surpass this conductivity. Our study highlights the benefits that can be gained by using *ab initio* approaches to guide material discovery.

## Methods

### Density functional theory calculations

All *ab initio* structure calculations were performed with calculations implemented in VASP^38^, using the projector augmented-wave method^39^. Calculations used the Perdew–Burke–Ernzerhof generalized-gradient approximation^40^. For energy calculations of NMPS structures, a Monkhorst–Pack *k*-point grid of 4 × 4 × 4 was used, for other competing phases, *k*-points were chosen such that *n*_kpoints_ × *n*_atoms_>1,000. The VASP pseudopotential set of Li (PAW_PBE Li 17Jan2003), Na (PAW_PBE Na 08Apr2002), Ge (PAW_PBE Ge 05Jan2001), Si (PAW_PBE Si 05Jan2001), Sn (PAW_PBE Sn_d 06Sep2000), P (PAW_PBE P 17Jan2003) and S (PAW_PBE S 17Jan2003) was used.

### Phase diagrams and stability limits

Phase diagrams are constructed using pymatgen^31^ by computing the lower convex hull of DFT computed energy per atom in composition space, similar to the work in ref. 25. Materials on this convex hull cannot decompose to a lower energy combination of phases and are therefore stable. Grand potential phase diagrams are constructed by computing the lower convex hull of the DFT computed grand potential (Φ[*c*,*μ*_Li_]=*E*[*c*]−*n*_Li_[*c*]*μ*_Li_, where *E*[*c*], *n*_Li_[*c*] and *μ*_Li_ are the DFT computed energy, lithium content and lithium chemical potential of composition *c*) in composition space. Anodic and cathodic stability limits are given by the maximum and minimum lithium chemical potentials for which a material (or its 0 K decomposition products in the case of metastable structures) is found to be stable.

### Conductivity simulations

We performed AIMD simulations under the Born–Oppenheimer approximation to determine Na diffusivity in the NMPS system using VASP^38^. Atom trajectories are calculated using Newtonian dynamics with Verlet integration in an NVT ensemble. A Nose–Hoover thermostat with a period of 40 timesteps (80 fs) was used for all simulations. Na-atom displacements are calculated with respect to the centre of mass of the framework (non-Na) atoms. Self-diffusivities from these simulations were calculated by fitting the Einstein relation of mean-squared displacements to time (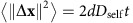), where *d* is the dimensionality, using tools implemented in the pymatgen software package^31^. Ionic conductivities taking into account correlations between Na-ions were calculated from the mean square displacement of the net Na-ion motion 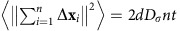. Inserting *D*_*σ*_ into the Nernst–Einstein equation is equivalent to using the Green–Kubo expression for ionic conductivity when Na-ions are the only mobile charge carriers^41^,^42^. The AIMD simulations were performed on a single unit cell of NMPS, with 50 ions (2 formula units). The volume and shape of the cells were obtained from the fully relaxed cells used for the energy calculations by enforcing tetragonal symmetry (equality of the *a* and *b* lattice parameters). The time step of the simulation was 2 fs. To reduce the computational cost of the calculation, forces were calculated using a single *k*-point. Temperatures were initialized at 300 K and scaled to the appropriate temperature over 1,000 time steps (2 ps), starting with the ground-state structure. Simulations between 600 and 900 K were 350,000 time steps (700 ps), and simulations above 900 K were 250,000 time steps (500 ps).

Calculation of the activation energy (*E*_a_) and extrapolation of results to room temperature was performed with an Arrhenius fit to the diffusivity data. The Haven ratio, *H*_r_, an indication of the cooperativity of ionic motion, is calculated from the ratio of *D*_self_ to the *D*_*σ*_ in each simulation.

### Ionic probability density

Na-ion probability densities were calculated from the AIMD simulations. After enforcing *P*4_2_/*nmc* symmetry, Na-ion positions relative to the centre of mass of the framework (P, M, S) atoms were smoothed using a Gaussian kernel with s.d. of 0.2 Å, and the resulting density visualized using Vesta^43^.

### Fractional occupancies

Fractional occupancies were calculated using a *k*-means clustering algorithm^44^, initialized with atomic positions from the structure of LGPS^26^. At each clustering step, the shortest distance (taking into account periodic boundary conditions) to each mean was calculated, and a linear assignment algorithm^45^ as implemented in pymatgen^31^ was used at each simulation time step to assign each Na-ion position to the nearest mean, ensuring that at most a single Na atom from each time step is assigned to any given mean. The resulting cluster sizes and centroids were used to define the occupancy and location of Na sites.

### Synthesis

Na_10_SnP_2_S_12_ was synthesized by mixing stoichiometric amounts of Na_2_S (Kojundo Chemical Laboratory Co. Ltd, 99%), P_2_S_5_ (Sigma-Aldrich Co., 99%) and SnS_2_ (Kojundo Chemical Laboratory Co. Ltd, 99.9%) with a planetary ballmill (380 r.p.m. for 17 h). The pelletized mixture was wrapped in gold foil and heated at 700 °C for 12 h in an evacuated quartz tube and slow-cooled down to room temperature for 99 h (approximately −0.1° min^−1^).

### X-ray diffraction

The X-ray diffraction pattern is obtained with Cu–K_α_ radiation (40 kV, 40 mA) from 10–90° 2θ with 0.03° step intervals.

### Conductivity measurement

Na-ion conductivity was measured with electrochemical impedance spectroscopy using an AUTOLAB PGSTAT30 (Metrohm Autolab, Utrecht) at 30, 40, 60 and 80 °C with a frequency ranging from 1 MHz to 100 mHz and an amplitude of 10 mV under normal pressure. An indium foil-blocking electrode was pressed onto both sides of the Na_10_SnP_2_S_12_ pellet (11.5 mm diameter and 0.75 mm thickness). The conductivity values were obtained from the Cole–Cole plot of the data.

## Additional information

**How to cite this article:** Richards, W. D. *et al*. Design and synthesis of the superionic conductor Na_10_SnP_2_S_12_. *Nat. Commun.* 7:11009 doi: 10.1038/ncomms11009 (2016).

## Supplementary Material

Supplementary Information Supplementary Figures 1-5 and Supplementary Tables 1-2

## Figures and Tables

**Table 1:** Ionic conductivity of cation-substituted compounds X_10_MP_2_S_12_.

Compound	DFT simulation		Experimental	
	*E*_a_ (eV)	Conductivity, 298 K (mS cm^−1^)	*E*_a_ (eV)	Conductivity, 298 K (mS cm^−1^)
Na_10_SiP_2_S_12_	0.229	10.28	NA	NA
Na_10_GeP_2_S_12_	0.270	3.50	NA	NA
Na_10_SnP_2_S_12_	0.317	0.94	0.356	0.4 (this work)
Li_10_SiP_2_S_12_	0.20	23 (ref. 20)	0.196	2.3 (ref. 21)
Li_10_GeP_2_S_12_	0.21	13 (ref. 20)	0.22–0.25	9–12 (refs 11, 22)
Li_10_SnP_2_S_12_	0.24	6 (ref. 20)	0.24–0.27	4–7 (refs 23, 24)

NA, not applicable. DFT simulation and experimental results on the sodium structures are from this work. Experimental and calculated values for the Li compounds are taken from the literature.

**Table 2:** Phase equilibria decomposition enthalpies and stability ranges for X_10_MP_2_S_12_.

Cation(X)	Cation(M)	Decomposition products	*E*_decomp_ (meV per atom)	Metal reduction (V versus metal anode)	Cathodic stability (V versus metal anode)	Anodic stability (V versus metal anode)
	Si	Na_4_SiS_4_+2 Na_3_PS_4_	13.6	0.80	1.25	1.77
Na	Ge	Na_4_GeS_4_+2 Na_3_PS_4_	7.2	1.10	1.25	1.70
	Sn	Na_4_SnS_4_+2 Na_3_PS_4_	7.1	1.09	1.25	1.82
	Si	Li_4_SiS_4_+2 Li_3_PS_4_	14.9	1.36	1.78	2.14
Li	Ge	Li_4_GeS_4_+2 Li_3_PS_4_	14.7	1.64	1.78	2.06
	Sn	Li_4_SnS_4_+2 Li_3_PS_4_	13.4	1.57	1.78	2.02
