# Supplementary material for: Design and synthesis of the superionic conductor Na10SnP2S12
Source: Nat Commun. 2016 Mar 17;7:11009. doi: 10.1038/ncomms11009 (PMC4800433; doi:10.1038/ncomms11009)
Supplement: Supplementary Information — Supplementary Figures 1-5 and Supplementary Tables 1-2 [file ncomms11009-s1.pdf]

Supplementary Figures

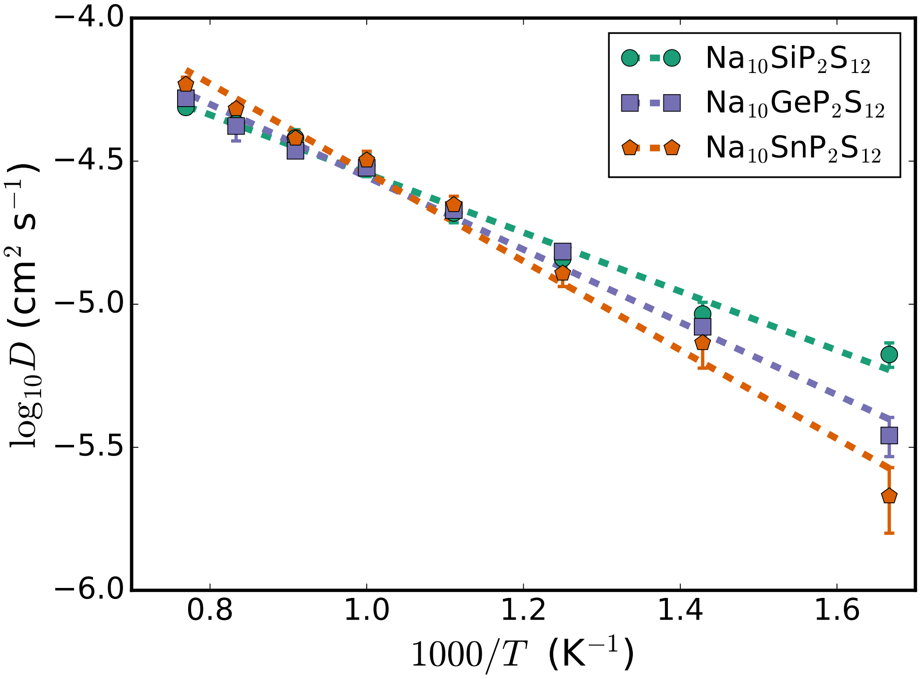

Supplementary Figure 1: Na self-diffusivity D_{self} in $Na_{10}SiP_2S_{12}$, $Na_{10}GeP_2S_{12}$, and $Na_{10}SnP_2S_{12}$ from AIMD simulation

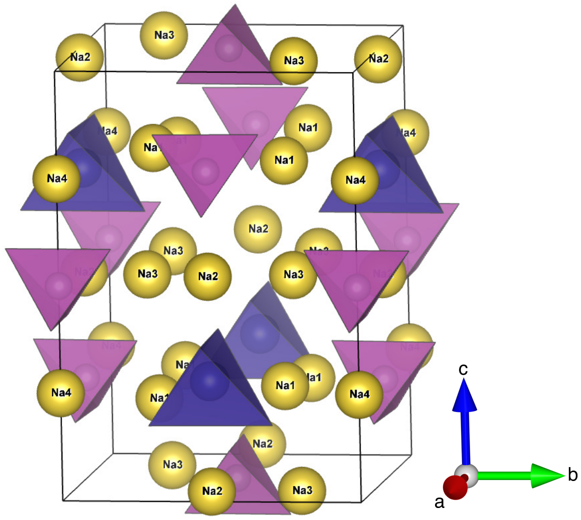

Supplementary Figure 2: $C222$ symmetry ground state ordering of Na atoms for the NMPS phases. Labels on the Na-atoms correspond to those of the $P4_2/nmc$ space group disordered structure.

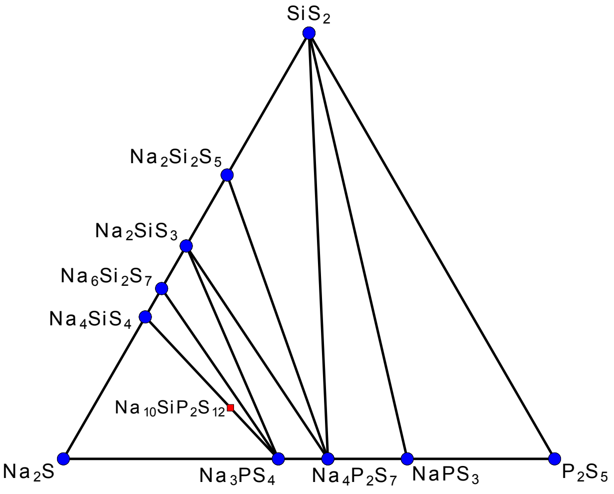

(a) (b)

Supplementary Figure 3: Pseudo-ternary 0K phase diagrams for the a) Na_2S-SiS_2-P_2S_5 and b) Na_2S-GeS_2-P_2S_5 chemical systems, computed from DFT energy calculations.

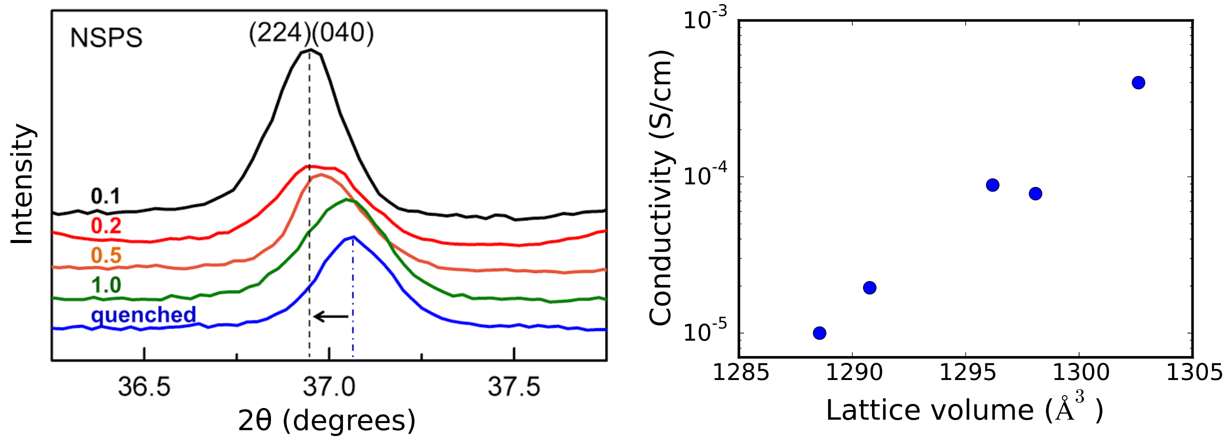

(a) (b)

Supplementary Figure 4: a) Main peak position for cooling rates of 0.1, 0.2, 0.5, and 1.0 K min^{-1}, and a quenched sample. Lattice volume increases as cooling rate is lowered b). Measured conductivity vs. lattice volume for these samples.

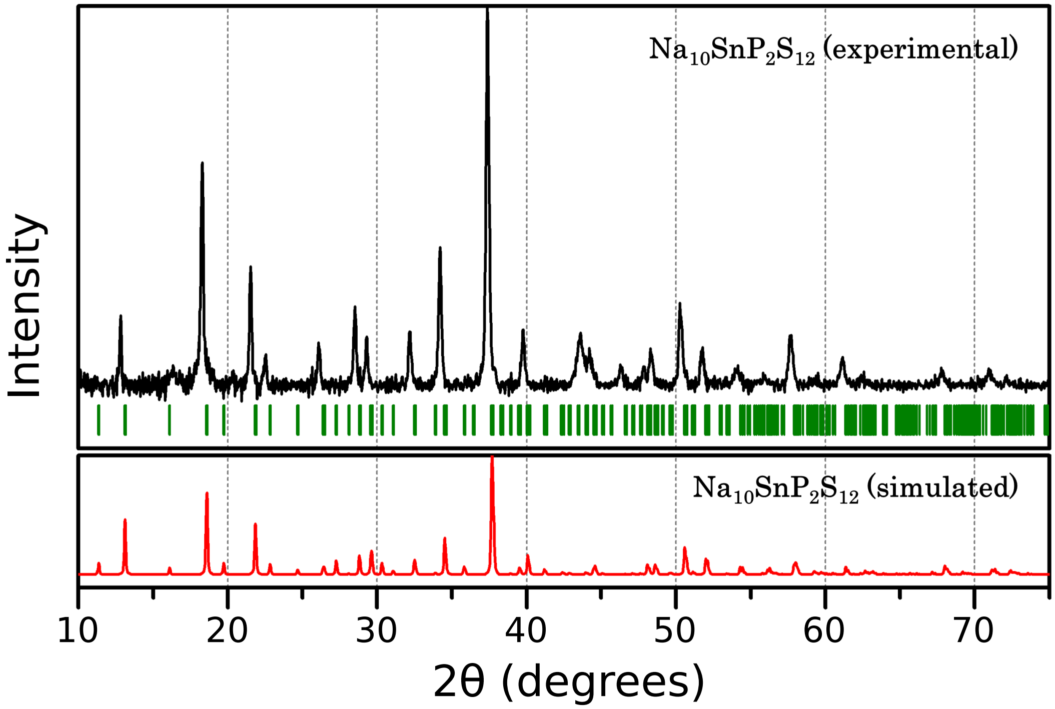

Supplementary Figure 5: Experimental and simulated XRD patterns of quenched $Na_{10}SnP_2S_{12}$.

Supplementary Tables

Compound	E_a (eV)	Self diffusivity D_{self} at 298 K (cm^2 s^{-1})
$Na_{10}SiP_2S_{12}$	0.204	1.13×10^{-7}
$Na_{10}GeP_2S_{12}$	0.252	2.97×10^{-8}
$Na_{10}SnP_2S_{12}$	0.307	6.94×10^{-9}

Supplementary Table 1: Calculated self diffusivity D_{self} of $Na_{10}MP_2S_{12}$ (M = Si, Sn, Al). Note that the differences between these activation energies and the activation energies of D_{corr} listed in table 1 are not statistically significant.

Cation (M)	a (Å)	c (Å)
Si	9.60	13.53
Ge	9.62	13.59
Sn	9.68	13.63

Supplementary Table 2: DFT calculated lattice parameters for tetragonal $Na_{10}MP_2S_{12}$ (M = Si, Ge, Sn).
